# Familial cases of a submicroscopic Xp22.2 deletion: genotype-phenotype correlation in microphthalmia with linear skin defects syndrome

**Published:** 2013-02-06

**Authors:** Sarah Vergult, Bart Leroy, Ilse Claerhout, Björn Menten

**Affiliations:** 1 Center for Medical Genetics, Ghent University Hospital & Ghent University, Ghent, Belgium; 2 Department of Ophthalmology, Ghent University Hospital & Ghent University, Ghent, Belgium

## Abstract

**Purpose:**

Microphthalmia with linear skin defects syndrome (MLS or MIDAS, OMIM #309801) is a rare X-linked male-lethal disorder characterized by microphthalmia or other ocular anomalies and skin lesions limited to the face and neck. However, inter- and intrafamilial variability is high. Here we report a familial case of MLS.

**Methods:**

A mother and daughter with MLS underwent a complete ophthalmological examination, and extensive imaging, including anterior segment pictures, corneal topography and keratometry, autofluorescence, infrared reflectance and red free images, as well as spectral-domain optical coherence tomography. The mother also underwent full-field flash electroretinography. In addition, high-resolution array comparative genomic hybridization analysis was performed in both as well as in the maternal grandparents of the proband.

**Results:**

Microphthalmia and retinal abnormalities were noted in the proband and the mother, whereas only the mother presented with scars of the typical neonatal linear skin defects. Array comparative genomic hybridization analysis revealed a 185–220 kb deletion on chromosome band Xp22.2 including the entire *HCCS* gene.

**Conclusions:**

The identification of a deletion including *HCCS* led to the diagnosis of MLS in these patients. Retinal abnormalities can be part of the ocular manifestations of MLS.

## Introduction

Microphthalmia with linear skin defects syndrome or MLS (OMIM #309801) is an embryonically male-lethal X-linked condition characterized by microphthalmia and linear, erythematous dermatological lesions. There is, however, high inter- and intrafamilial variability. Until now, 61 patients have been described of whom 11 cases were familial (Appendix 1) [1-41]. Of these 61 patients, ten were reported to have eye anomalies without skin lesions, and four presented with skin defects without eye anomalies. In most of the cases, the syndrome is caused by deletion of a region on chromosome band Xq22.2 containing three genes *MID1*, *HCCS*, and *ARHGAP6* of which *HCCS* is the only gene entirely contained within this interval [42,43]. At first, *ARHGAP6*, which encodes a rho GTPase activating protein (GAP), was considered the most likely candidate gene [16]. However, Wimplinger et al. recently reported three patients with MLS with a de novo mutation in the *HCCS* gene, indicating that *HCCS* is the causal gene for MLS [29,31]. This gene encodes a mitochondrial enzyme, the holocytochrome c synthase, which covalently links a heme group to the apoprotein of cytochrome *c* and *c_1_*, resulting in the formation of holocytochrome *c* and *c_1_.* Holocytochrome *c* plays an essential role in oxidative phosphorylation in the mitochondrial respiratory chain [44,45]. Here we report the first familial cases in which unilateral microphthalmia with linear skin defects in the mother and isolated microphthalmia in the daughter are caused by a microdeletion spanning the entire *HCCS* gene and part of *ARHGAP6*.

## Methods

### Clinical examination

**Figure 1:**
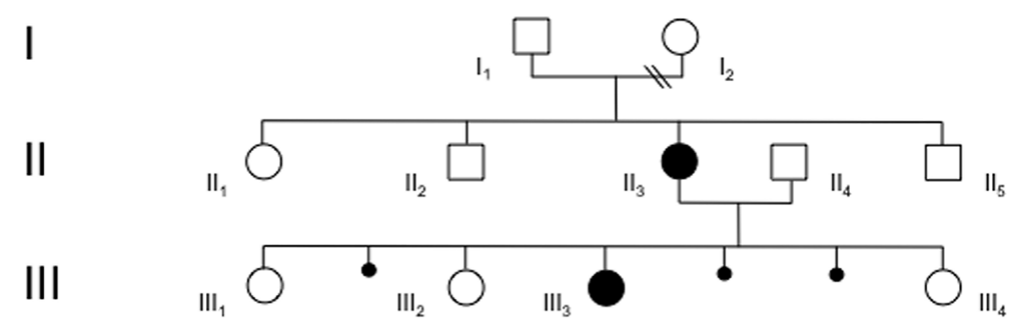
Pedigree of family with microphthalmia with linear skin defects syndrome. Open circles represent healthy females, filled circles are affected females, squares are healthy males, and small filled circles represent spontaneous abortions in the first trimester of pregnancy; the sex of the fetuses of the latter was unknown.

Informed consent was obtained from all subjects, including the grandparents following the Tenets of Helsinki. This study was approved by the ethics committee of Ghent University Hospital. The proband is a 12-year-old girl, while her mother is 45 (Figure 1). Both patients are affected with MLS and underwent a complete ophthalmological examination and extensive imaging, with anterior segment pictures, corneal topography and keratometry (Pentacam, Oculus, Wetzlar, Germany, and Nidek KM-500, Aichi, Japan), autofluorescence, infrared reflectance and red free images, as well as spectral-domain optical coherence tomography (HRA2 and Spectralis, Heidelberg Engineering, Heidelberg, Germany) in both, and electroretinography (Retiport, Roland Consult, Brandenburg an der Havel, Germany) in the mother. In addition, a general medical examination, including blood pressures, cardiac and pulmonary auscultation, weight, height and head circumference measurements, was performed.

### Cytogenetic and molecular analysis

Conventional karyotyping was performed with G-banding using standard procedures on short-term lymphocyte cultures. For array comparative genomic hybridization (CGH) analysis, DNA was isolated from the total blood using the QIAamp DNA Blood Mini Kit (Qiagen, Venlo, The Netherlands) according to the manufacturer’s instructions. Copy number profiling was performed on 180 K oligonucleotide arrays (Agilent Technologies, Diegem, Belgium) according to the manufacturer’s instructions with minor modifications as described [46].

### X-inactivation assay

Examination of the methylation pattern at the androgen receptor (*AR*) locus was performed on genomic DNA isolated from blood leucocytes according to Allen et al. [47]. More specifically, analysis of a polymorphic CAG repeat in the first exon of the human androgen-receptor gene was used to determine the X-inactivation state. The methylation status of a specific restriction enzyme site in the proximity of the polymorphic CAG short tandem repeat correlates with X-chromosome inactivation [47].

### Parental testing

Parental testing was performed on genomic DNA isolated from the total blood using the Powerplex 16 System (Promega, Leiden, The Netherlands) according to the manufacturer’s instructions.

## Results

### Phenotype

**Figure 2:**
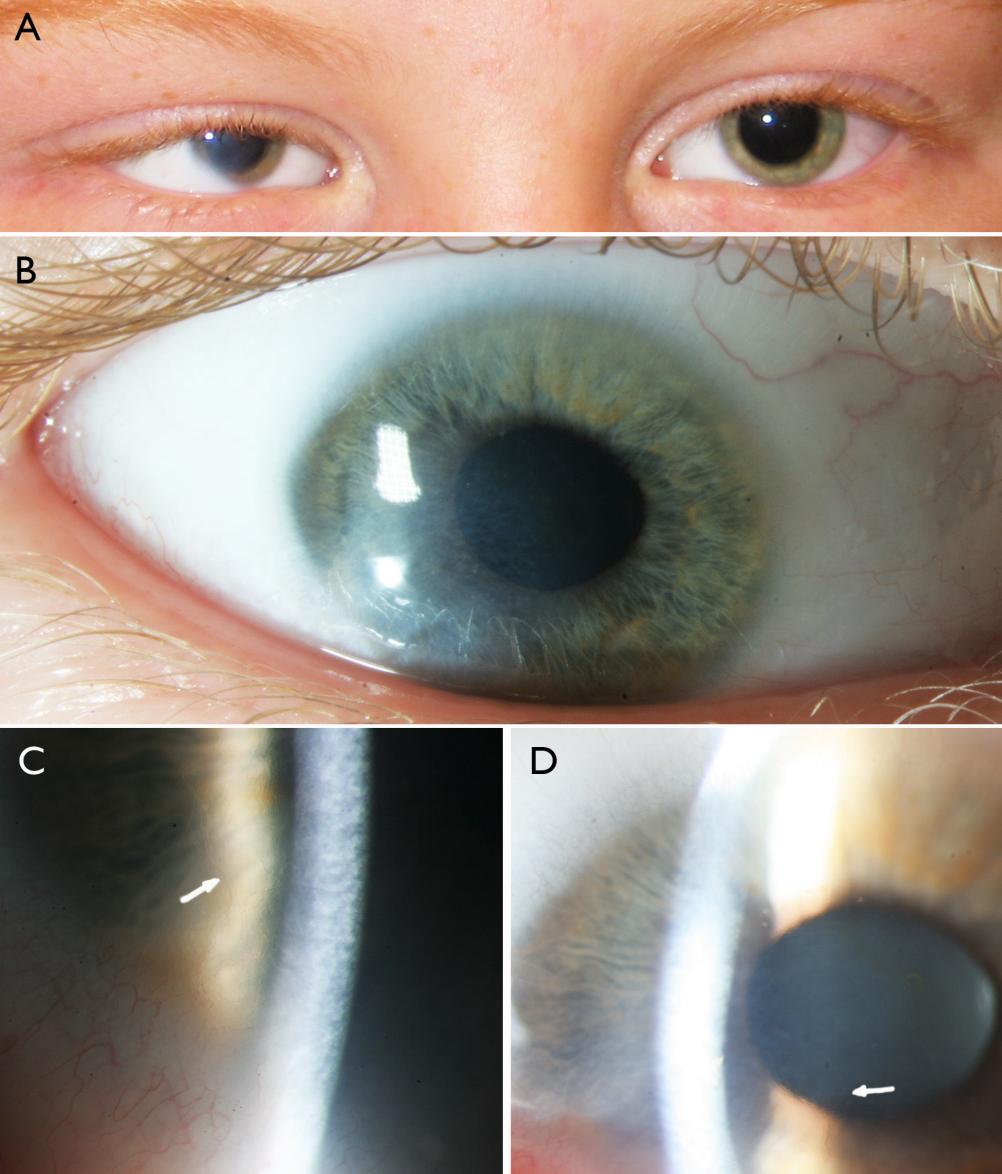
All images from the proband. **A**: Microphthalmic right eye and clinically normal left eye; notice esotropia and corneal opacification of the right eye. **B**: Right eye with corneal opacification in central and inferotemporal areas. **C**: Detail of inferotemporal area of right eye; notice patent peripheral corneal vessels (arrow) in area of reticular endothelial abnormalities and relative stromal opacification. **D**: Detail of central area of right eye; notice central corneal ghost vessels (arrow) in area of corneal opacification.

The proband shows esotropia, with microphthalmia of the right eye, which is predominantly due to a reduced anterior chamber depth (Appendix 2). The right eye also showed microcornea, with a normal aspect of the cornea in the superior quarter, but with posterior bulging and thinning of the cornea in the central and inferotemporal areas, combined with a reticular golden white stromal haze and endothelial abnormalities in a honeycomb pattern posterior to an undulating Descemet membrane, as well as patent intrastromal corneal blood vessels inferotemporally, continuous with ghost vessels more centrally (Figure 2). The iris stroma was slightly hypoplastic, most pronounced in the inferior and temporal area, with an anterior synechia at seven o’clock. Fundoscopy showed no gross abnormalities in the microphthalmic right eye. The left eye was entirely normal apart from cornea plana (Appendix 2).

**Figure 3:**
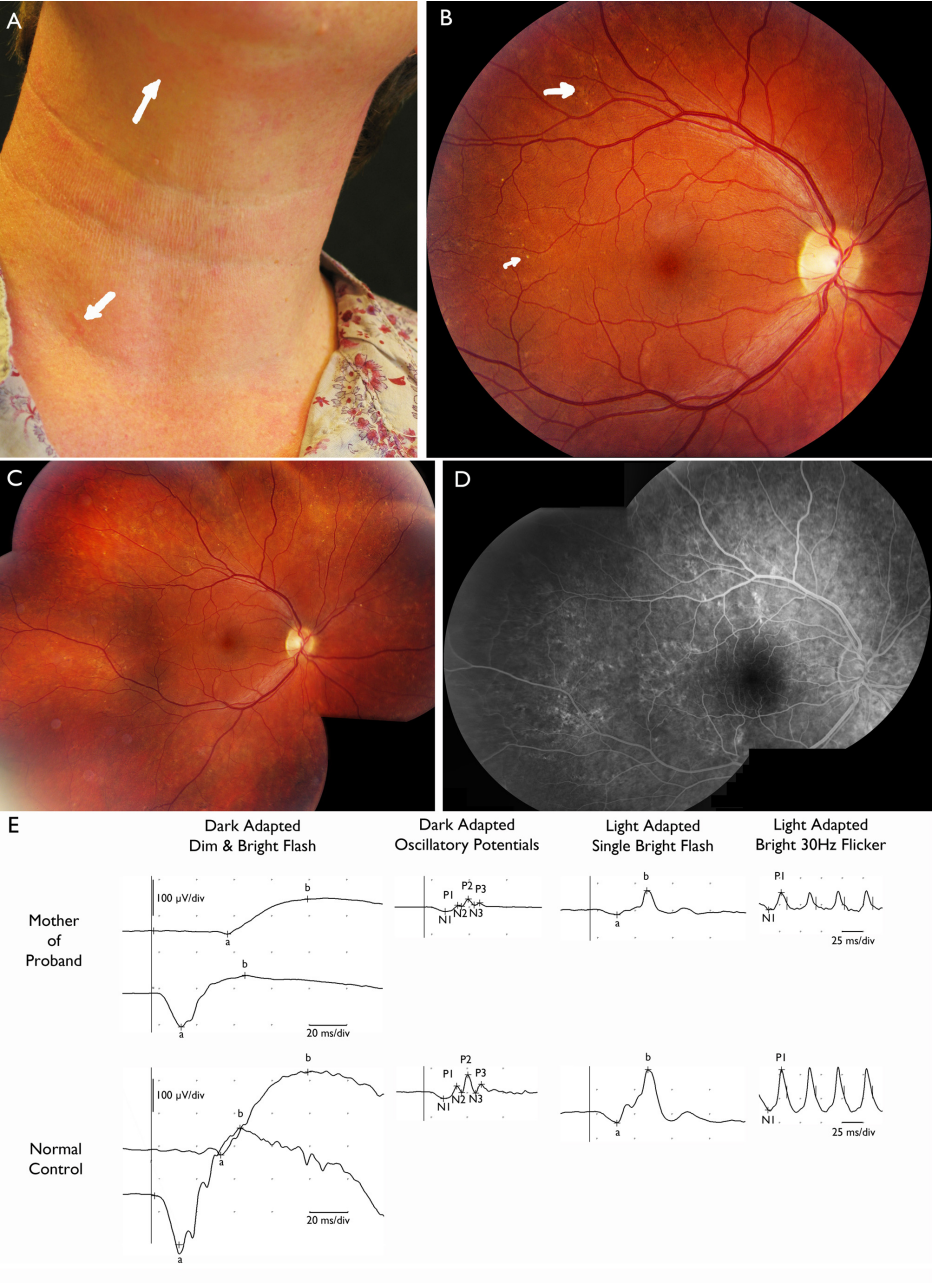
All images from the mother of the proband. **A**: Multiple erythematous scars in neck (two indicated by arrows); lesions had aspect of hemorrhaging incisions in the neonatal period. **B** and **C**: Fundus abnormalities with white, drusenoid deposits and reticular RPE abnormalities in the macula (**B**: Small and large arrow respectively), and whole fundus of left eye (**C**), with reticular abnormalities with alternating hypo- and hyperpigmentation of retinal pigment epithelium (RPE). **D**: Hyper- and hypointense areas on fluorescein angiography in keeping with RPE abnormalities described under A. **E**: Full-field flash electroretinography of the right eye (top traces); traces at bottom are from normal age-matched control for purposes of comparison. Notice overall reduction of retinal function for rod-specific, combined rod-cone, and cone-specific responses to about three-fifths of normal amplitudes, without significant delay in responses. Retinal function is thus reduced in keeping with the reduced total surface area of the retina, but the remaining areas function normally, suggesting this is not progressive retinal degeneration.

The mother of the proband had her left eye enucleated at an early age because of severe microphthalmia, to avoid consequent orbital hypoplasia, and has since worn an ocular prosthesis. The anterior segment of her right eye was normal, whereas fundoscopy, fluorescein angiography, and optical coherence tomography showed patches of outer retinal abnormalities with white dots and pigment epithelial alterations temporal from the macula and into the whole periphery (Figure 3). Electroretinography showed subnormal overall retinal function with all amplitudes reduced to approximately three fifths of normal values, albeit without delayed peak times (Figure 3). Whereas the mother had scars in the neck area consequent upon neonatal skin lesions typical of MLS (Figure 3), the skin of the proband was entirely normal.

A general medical and cardiological examination was entirely normal in both patients. The mother completed higher education, whereas the daughter is successful in high school.

In keeping with the male lethality of the MLS, the mother of the proband had three additional healthy daughters, and suffered three spontaneous abortions in the first trimester of pregnancy (Figure 1). Although never proven, these might have been male fetuses or severely affected female fetuses.

### Genotype

Conventional karyotyping revealed a normal female karyotype, 46,XX, in the proband and her mother. Subsequent array CGH analysis revealed submicroscopic deletion of 185–220 kb on chromosome band Xp22.2 in the proband and the mother. Neither the maternal grandmother nor the maternal grandfather of the proband carried the Xp22.2 deletion, indicating that the deletion arose de novo in the mother. Paternity testing confirmed that both grandparents are the biologic parents of the mother and that the mother is indeed the biologic mother of the proband.

**Figure 4:**
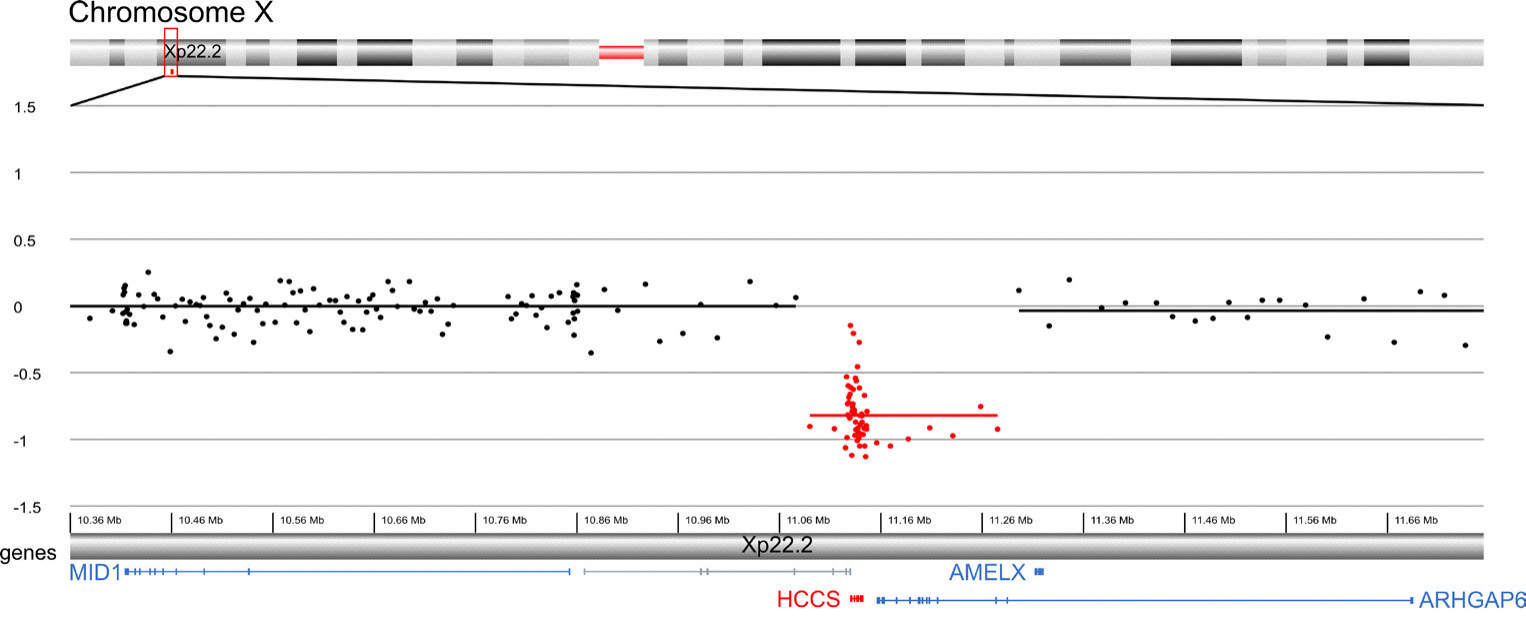
Overview of the 185–220 kb deletion on chromosome band Xp22.2 from the mother and the proband. Data shown are from the proband. Genes in and surrounding this region are shown at the bottom. The HCCS gene is highlighted in red; all other coding genes in the proximity are shown in blue. Noncoding sequences such as long noncoding RNAs are given in gray. Genomic coordinates are given in GRCh37 (hg build 19).

The deleted region contains the entire *HCCS* gene and partially spans *ARHGAP6* (Figure 4). The telomeric breakpoint lies in the intergenic region between *MID1* and *HCCS*. The centromeric breakpoint lies in *ARHGAP6*. This deletion has not yet been reported by the Toronto Database of Genomic Variants. The data of the mother (Patient 255,415) and the proband (Patient 255,414) were submitted to the DECIPHER database (Database of Chromosomal Imbalance and Phenotype in Humans using Ensembl Resources).

X-inactivation testing revealed complete skewing in the mother and the proband. The chromosome containing the deletion is likely the inactive one in the mother and the proband.

## Discussion

Most of the previously reported MLS cases are sporadic although a few families with multiple affected individuals have been reported [2,18,20,29,30]. Here we report on the first familial cases in which the deleted region spans the entire *HCCS* gene and part of the *ARHGAP6* gene.

The proband reported here presents with unilateral microphthalmia without skin lesions, whereas the mother had scars from neonatal linear skin defects in the neck region. The pattern of ocular abnormalities seen in the microphthalmic right eye of the proband suggests the microphthalmia is due to the predominant involvement of the anterior segment of the eye (anterior microphthalmia). In addition, the central and inferotemporal opacities of the right cornea in the proband represent the mild end of the spectrum of corneal opacification, of which classic sclerocornea is the extreme end. Corneal opacification has been described in 15 patients, and sclerocornea in 17 patients reported with ocular involvement [3,6,11,15,19,24,29,30].

Only four reports mention retinal abnormalities in MLS patients, varying from chorioretinopathy over abnormalities of the retinal pigment epithelium (RPE) and foveal hypoplasia in eyes that also show microphthalmia [7,19,21], as well as multiple patches of hypopigmentation of the RPE in an otherwise normal eye [24]. Fundoscopy and fluorescein angiography of the right eye of the mother of the proband, showed white, drusenoid deposits and linear RPE abnormalities with hyper- and hypopigmentation in the peripheral macula and midperipheral retina (Figure 3). These findings, combined with the reduced overall retinal function as measured with electroretinography (Figure 3), suggest that retinal abnormalities may be the only ocular manifestation of MLS in an otherwise normal eye.

Although the characteristics of MLS are microphthalmia and linear skin lesions, there is inter- and intrafamilial phenotypic variability. Ocular and skin anomalies are present in most of the reported patients (47/61). A small number do not present with ocular manifestations (4/61), and others do not have skin anomalies (11/61; Appendix 1). It has been proposed that X-inactivation may play a role in the phenotypic variability in affected female patients. In 16/21 of the analyzed female individuals with MLS, skewed X-inactivation of the abnormal X chromosome was detected (Appendix 1). As it is suggested that X-inactivation ratios may vary between different tissues within an individual [48,49], the inter- and intrafamilial phenotypic variability is probably caused by differing degrees of skewing. Morleo et al. hypothesized that a milder phenotype or the total absence of MLS clinical manifestations may be due to a totally skewed X-inactivation that forces preferential activation of the unaffected X [50]. Here complete skewing was noted in the mother and daughter on blood leukocytes for which the X chromosome containing the deletion is likely the inactive one [29,30,50,51]. The clinical manifestations in the daughter are less severe than in the mother. It is indeed likely that the severity of the phenotype is more related to the tissue-specific degree of X-inactivation of the X-chromosome with either a mutation in, or a deletion of HCCS, than the effect of the mutation on the protein product itself. Since the proband does not have skin lesions, and the scars resulting from the neonatal skin defects in the mother are minimal, the two were not diagnosed with MLS at first.

Interestingly, Wimplinger et al. reported a de novo loss-of-function mutation in *HCCS* in a female with bilateral microphthalmia and severe sclerocornea, suggesting that *HCCS* is a candidate gene for severe eye malformations [31].

To further unravel the role of *HCCS* in the development of the eye, more patients with severe eye malformations need to be screened, and functional studies are required. It will also be interesting to see whether the microphthalmia is more commonly of an anterior type in MLS.

The use of array CGH in our patients has proven to be useful as the 185–220 kb deletion in these patients would have never been detected with conventional karyotyping. Most of the previous cases had larger cytogenetically visible deletions or translocations. Recently, Balikova et al. highlighted the importance of array CGH in diagnostics for patients with congenital eye malformations [52]. Using this technique, the phenotypic spectrum associated with the deletion of *HCCS* can be further broadened. In conclusion, we report on a family with two cases of unilateral microphthalmia of whom only one showed scars of neonatal linear skin lesions with a 185–220 kb microdeletion containing *HCCS* and *ARHGAP6*, supporting the hypothesis that *HCCS* is a candidate gene for severe eye malformations.

## Appendix 1.

Overview of all patients reported with MLS. Familial cases are highlighted in bold. Patients reported in abstracts or patients for whom no aberration on the X-chromosome was detected were left out of this table. NA=not applicable, ND=not determined. To access the data, click or select the words “Appendix 1.” This will initiate the download of a pdf file.

## Appendix 2.

Ocular measurements of proband and her mother and normal values for comparison. To access the data, click or select the words “Appendix 2.” This will initiate the download of a pdf file.
